# Physical activity and phubbing behavior in Chinese college students: the mediating role of self-control and the moderating role of gender

**DOI:** 10.3389/fpsyg.2025.1613727

**Published:** 2025-07-10

**Authors:** Yue Li, Shanshan Yin, Huijuan Yi, Hao Zhu

**Affiliations:** ^1^Ministry of Public Sports, Taizhou University, Taizhou, China; ^2^Jiangsu Food & Pharmaceutical Science College, Huai'an, China; ^3^School of Physical Education and Humanities, Nanjing Sport Institute, Nanjing, China

**Keywords:** physical activity, phubbing behavior, self-control, college students, moderated mediation

## Abstract

**Objective:**

The prevalent adoption of smartphones has given rise to widespread phubbing behavior among college students, characterized by excessive smartphone use in social settings. However, research investigating behavioral intervention strategies to mitigate phubbing behavior remains notably scarce. In the present study, we examined the mediating mechanism of self-control and the moderating role of gender between physical activity and phubbing behavior in college students.

**Methods:**

This study was conducted involving 1,340 college students using the Physical Activity Rating Scale-3, Phubbing Scale, and Self-Control Scale, respectively. Data analysis by using SPSS 27.0, including mediation analysis and moderating analysis.

**Results:**

Physical activity had a direct negative influence on phubbing behavior [*β* = −0.279, 95% CI (−0.331, −0.227)], while self-control acted as a mediator in this indirect relationship [*β* = −0.123, 95% CI (−0.150, −0.098)]. Additionally, female college students’ participation in physical activity had a stronger impact on improving self-control and reducing phubbing behavior compared to male students.

**Conclusion:**

The current research indicated that physical activity constituted an effective intervention for preventing and reducing phubbing behavior in college students, which could either directly affect college students’ phubbing behavior or indirectly through the mediating variable of self-control. Furthermore, gender moderated the effect of physical activity, self-control, and phubbing behavior, with female students’ physical activity participation exhibiting stronger predictive effects on enhancing self-control and alleviating phubbing behavior compared with male college students.

## 1 Introduction

Smartphones have profoundly transformed human lifestyles in recent years, and have provided unprecedented convenience for interpersonal communication. However, the increasing time invested in smartphone usage has concurrently exerted adverse effects on face-to-face interactions. College students are generally equipped with smartphones and have considerable autonomy in time management. Research findings have shown that college students engage with their smartphones for roughly 6 h each day while nearly 69% of these students display characteristics of high-risk smartphone addiction (Turki et al., 2023). The extensive use of smartphones leads to both smartphone addiction and a new social impairment called “phubbing” behavior where people ignore their companions at social gatherings because they pay more attention to their smartphones rather than engaging in conversation (Chotpitayasunondh and Douglas, 2016). Individuals subjected to phubbing behavior have a diminished sense of belonging due to perceived neglect, thereby triggering negative emotional and behavioral responses [e.g., undermining relationship satisfaction (Chotpitayasunondh and Douglas, 2018), decreasing life satisfaction (Karaman and Arslan, 2024) and hindering intimacy (Wang Q. et al., 2024; Wang X. et al., 2024)], which could escalate into clinical symptoms including anxiety disorders and depressive symptomatically (Ivanova et al., 2020; Li et al., 2024). Phubbing behavior has become a pervasive phenomenon among college students, with its negative impact on mental health has attracted widespread attention in academic circles. Nevertheless, previous studies have predominantly focused on delineating predictive factors and consequences of phubbing behavior, while systematic exploration of behavioral intervention strategies remains markedly underdeveloped, especially the absence of research on the mechanisms of potential protective factors.

Numerous studies have consistently demonstrated that regular engagement in physical activity contributes to enhancing physiological well-being while simultaneously representing a viable intervention strategy for addictive disorders. Empirical evidence has revealed that this vital benefit mechanism encompasses diverse forms of addiction, including but not limited to nicotine dependence (Ni et al., 2022), substance abuse (Martinez-Calderon et al., 2023), and smartphone addiction (Ke et al., 2024). Phubbing behavior is a novel social maladaptation closely associated with smartphone addiction, emerging as individuals prioritize device interaction over real-world engagement. Grounded in the Uses and Gratifications Theory, numerous studies on regular active engagement in physical activity have confirmed the benefits of not only reducing screen time, enhancing interpersonal relationships, and alleviating emotional stress, but also decreasing susceptibility to smartphone addiction by fulfilling individuals’ innate psychological needs and facilitating internalization of self-regulatory behaviors (Cardenas et al., 2009; Zhong et al., 2021). Smartphone addiction exhibited a significant correlation with participation in physical activities, while phubbing behavior also demonstrated associations with physical engagement. One study has indicated that adolescents exhibiting frequent phubbing behavior demonstrate weaker motivation for physical participation (Kim et al., 2015). Another study by Shen and Wei (2022) established that adolescent phubbing behavior significantly predicts reduced participation in physical activity 3 months later. Phubbing behavior exerted a direct impact on individuals’ participation in physical activities. Given the established consensus that physical activity is a positive behavioral modality capable of suppressing maladaptive tendencies and demonstrates particular efficacy in alleviating smartphone addiction. Therefore, it is reasonable to presume that physical activity has a bidirectional influence in relation to phubbing behavior. In view of these findings, we hypothesize that physical activity negatively predicts college students’ phubbing behavior (*H1*).

Self-control is conceptualized as the ability to consciously regulate individuals’ overt behavioral reactions and internal affective and cognitive processes (Hofmann et al., 2009). As a positive psychological attribute, it constitutes a critical determinant of personal success, with its developmental trajectory demonstrating lifelong plasticity potential that has attracted substantial research interest. Deficits in self-control capacity exhibited robust associations with diverse personal adversities [e.g., addiction (Yang et al., 2022), delinquent (Boisvert et al., 2012), academic failure (Zhang et al., 2024)]. Individuals with robust self-control capacity demonstrate enhanced behavioral regulation, consequently manifesting lower phubbing behavior during social interactions. Evidence from psychological studies has substantiated the inhibitory role of self-control capacity in relation to phubbing behavior, with heightened self-control demonstrating a robust inverse correlation with the prevalence of such phubbing behavior (Davey et al., 2018). Furthermore, building on the theoretical framework of the Strength Model of Self-Control. It suggested that people who active participation in physical activity can augment psychological energy reservoirs, thereby enhancing self-control ability. Substantial empirical research has consistently identified that college students who exhibit higher physical activity levels demonstrate superior self-control relative to their less active counterparts (Kennett et al., 2009; Schöndube et al., 2017; Yu and Mu, 2024). Prior studies have consistently indicated that improved self-control is an important influence in preventing the emergence of phubbing behavior among college students, and existing empirical investigations have also demonstrated a significant beneficial impact of physical activity on self-control. However, the interrelationships among physical activity, self-control, and phubbing behavior remain insufficiently elucidated in current academic research. Building upon that foundation of research, it is plausible to posit that active engagement in physical activities potentially mitigate phubbing behavior among college students through the enhancement of self-control capabilities. Consequently, the following research hypothesize is advanced that self-control mediates the association between physical activity and phubbing behavior (*H2*).

Additionally, research findings have demonstrated that physical activity and phubbing behavior vary significantly by gender, which are shaped by individual lifestyle habits, personality traits, and gender role orientations. Specifically, exhibited significantly greater engagement in physical exercise compared to their female counterparts, whereas female college students displayed a higher propensity for phubbing behavior (Azevedo et al., 2007; Balta et al., 2018; Buizza et al., 2022; Wang Q. et al., 2024; Wang X. et al., 2024). Moreover, existing research has also demonstrated gender-based variations in self-control capacities, yet no consensus has been reached on this matter (Chui and Chan, 2016; Hamama and Hamama-Raz, 2021). Therefore, investigating the potential moderating effects of gender in the relationship between physical activity, self-control, and phubbing behavior in college students constitutes a further focus of this research. In summary, we hypothesize that the direct and indirect relationships between physical activity and phubbing behavior via self-control are moderated by gender (*H3*). On the basis of these hypotheses, we construct a comprehensive conceptual model of the potential effects of physical activity and self-control on college students’ phubbing behavior by integrating mediating pathways and moderating factors.

## 2 Materials and methods

### 2.1 Participants

To maximize sample diversity, this study recruited a total of 1,400 participants from three universities strategically located in the southern, central, and northern regions of Jiangsu Province, China. The survey was conducted through both online and offline formats, administered by professionally trained staff following standardized protocols. Each participant received detailed information about the research objectives and procedures before data collection. After excluding incomplete or inaccurate responses (e.g., identical answer patterns or abnormally short completion times), a total of 1,340 valid questionnaires were retained, which constituted an effective response rate of 95.71%. The study sample included 588 male participants who formed 43.88% of the total sample while 752 female participants made up 56.12%, and age was 19.5 ± 0.88. The geographical distribution of respondents showed 772 participants (57.61%) from rural regions and 568 respondents (42.39%) from urban areas. The study’s cohort consisted of 506 only-child individuals who represented 37.76% and 834 non-only children which represented 62.24%.

### 2.2 Measures

#### 2.2.1 Physical activity rating scale-3

The revised version of the Physical Activity Rating Scale-3 (PARS-3) developed by Liang (1994) served as the evaluation tool for measuring physical activity participation in college students. The PARS-3 employed a 5-point Likert-type system comprising three dimensions: namely exercise duration, frequency, and intensity. Total scores = intensity × (time-1) × frequency, and producing results between 0 and 100. Based on established criteria (Xia et al., 2018), we divided participants’ physical activity into three distinct activity levels which include low-level physical activity (scores ≤19), moderate-level physical activity (20–42), and high-level physical activity (scores ≥43). People who scored higher demonstrated stronger participation in physical activity. The Cronbach’s *α* for the PARS-3 was 0.73.

#### 2.2.2 Self-control scale

The revised version of the Self-Control Scale (SCS) developed by Tan and Guo (2008) served as the evaluation tool for measuring self-control in college students. The SCS employed a 5-point Likert-type system comprising five dimensions: namely health habits, entertainment moderation, impulse control, resistance to temptation, and academic performance. Higher scores reflected greater self-control capacity. The Cronbach’s *α* for the SCS was 0.90.

#### 2.2.3 Phubbing behavior scale

The revised version of the Phubbing Behavior Scale (PS) developed by Hui et al. (2022) served as the evaluation tool for measuring phubbing behavior in college students. The PS employed a 5-point Likert-type system comprising two dimensions: namely communication disturbance and smartphone addiction. Elevated scores signified greater severity of phubbing behavior. The Cronbach’s α coefficient for the PS was 0.86.

### 2.3 Statistical analysis

This study applied the SPSS 27.0 for data analysis and testing. Firstly, for standardized data, gender differences in each main variable were investigated using the Mann–Whitney U-test. Secondly, correlations between variables were analyzed using Pearson correlation analysis. Finally, moderated mediation model analyses were conducted using PROCESS 4.1 (Model 4 and Model 59) developed by Hayes (2013) to explore the mediating mechanism of self-control and the moderating effect of gender, respectively. Bootstrapping with 5,000 resamples was applied to evaluate mediated moderation effects, while moderation patterns were visualized through slope plots. Statistical importance was set at *p* ≤ 0.05 for all analyses. Additionally, through the implementation of anonymous measurement protocols and the inclusion of partial reverse-scored items to control for common method bias. Harman’s single-factor test revealed that seven factors with a trait root greater than 1.0, and the first common factor interpretation percentage was 27.29% (< 40% threshold) (Podsakoff et al., 2003). Therefore, these results collectively indicated that there were no serious common methodological biases in this investigation.

## 3 Results

### 3.1 Gender differences in each main variables

Gender differences in each main variables were investigated using the Mann–Whitney U-test. As presented in Table 1, with male college students’ physical activity (22.67 ± 21.35,) and self-control (65.19 ± 10.85) showed higher than female college students’ (11.76 ± 13.10, 60.92 ± 11.10), and female college students’ phubbing behavior (26.30 ± 6.55) exhibited higher than males college students’ (25.14 ± 5.18).

**Table 1:** Mann–Whitney *U*-test of gender (*M* ± SD).

	PA	SC	PB
Mann–Whitney U	150233.000	172285.500	194299.000
Wilcoxon W	433351.000	455413.500	367395.000
*Z*	−10.11	−6.945	−3.825
Asymptotic saliency (two-tailed)	< 0.001	< 0.001	< 0.001
Male	22.67 ± 21.35	65.19 ± 10.85	25.14 ± 5.18
Female	11.76 ± 13.10	60.92 ± 11.10	26.30 ± 6.55

PA, Physical Activity; SC, Self-Control; PB, Phubbing Behavior; M, mean; SD, standard deviation.

### 3.2 Descriptive statistics and correlation analysis

As presented in Table 2, the correlation analysis results showed a statistically significant positive association between physical activity and self-control (*r* = 0.378, *p* < 0.001). Conversely, physical activity (*r* = −0.394, *p* < 0.001) and self-control (*r* = −0.443, *p* < 0.001) were negatively correlated with phubbing behavior.

**Table 2:** Descriptive statistics and correlation analysis.

Variables	*M*	SD	1	2	3
1. PA	16.55	18.04	1		
2. SC	62.79	11.19	0.378***	1	
3. PB	25.79	6.25	−0.394***	−0.443***	1

PA, Physical Activity; SC, Self-Control; PB, Phubbing Behavior; M, mean; SD, standard deviation; all variables in the model were standardized; *p < 0.05; **p < 0.01; ***p < 0.001.

### 3.3 Mediation and moderation analysis

#### 3.3.1 Testing the mediate role of self-control

The mediation effect test was conducted utilizing PROCESS (Model 4) and the findings are presented in Table 3. After controlling for covariates (e.g., gender, whether participants were only children, and growth environment), the overall regression model achieved statistical significance (*R*^2^ = 0.395, *F* = 61.775, *p* < 0.001). Physical activity positively predicted self-control (*β* = 0.353, *p* < 0.001). Both physical activity (*β* = −0.279, *p* < 0.001), and self-control (*β* = −0.348, *p* < 0.001) negatively predicted phubbing behavior. Subsequently, to examine the mediating mechanism, we employed Bootstrap approach with 5,000 repeated samplings for statistical verification. As presented in Table 4. The analysis revealed that physical activity exerted a significant direct influence on phubbing behavior [*β* = −0.279, 95% CI = (−0.331, −0.227)], contributing 69.40% of the total effect. Furthermore, self-control mediated an indirect pathway [*β* = −0.123, 95% CI (−0.150, −0.098)], and this mediating effect accounted for 30.60% of the total effect. Therefore, hypotheses 1 and 2 were supported.

**Table 3:** Regression analysis of the mediation model.

Criterion	Predictors	*R*	*R* ^2^	*F*	*β*	*t*
SC		0.387	0.150	58.660		
	PA				0.353	13.366***
	Gender				−0.084	−3.120**
	If the only-child				−0.001	−0.010
	Growth environment				−0.007	−0.272
PB		0.509	0.259	93.450		
	PA				−0.279	−10.632***
	SC				−0.348	−13.635***
	Gender				−0.056	−2.249*
	If the only-child				−0.003	−0.112
	Growth environment				−0.013	0.522

PA, Physical Activity; SC, Self-Control; PB, Phubbing Behavior; *p < 0.05; **p < 0.01; ***p < 0.001.

**Table 4:** Mediation effect of self-control.

Phubbing behavior	Effect	BootSE	BootLLCI	BootULCI	Effect percentage
Total effect of PB	−0.402	0.026	−0.454	−0.351	
Direct effect of PB	−0.279	0.026	−0.331	−0.227	69.40%
Mediate effect of SC	−0.123	0.013	−0.150	−0.098	30.60%

PA, Physical Activity; SC, Self-Control; PB, Phubbing Behavior.

#### 3.3.2 Moderating role of gender

**Figure 1:**
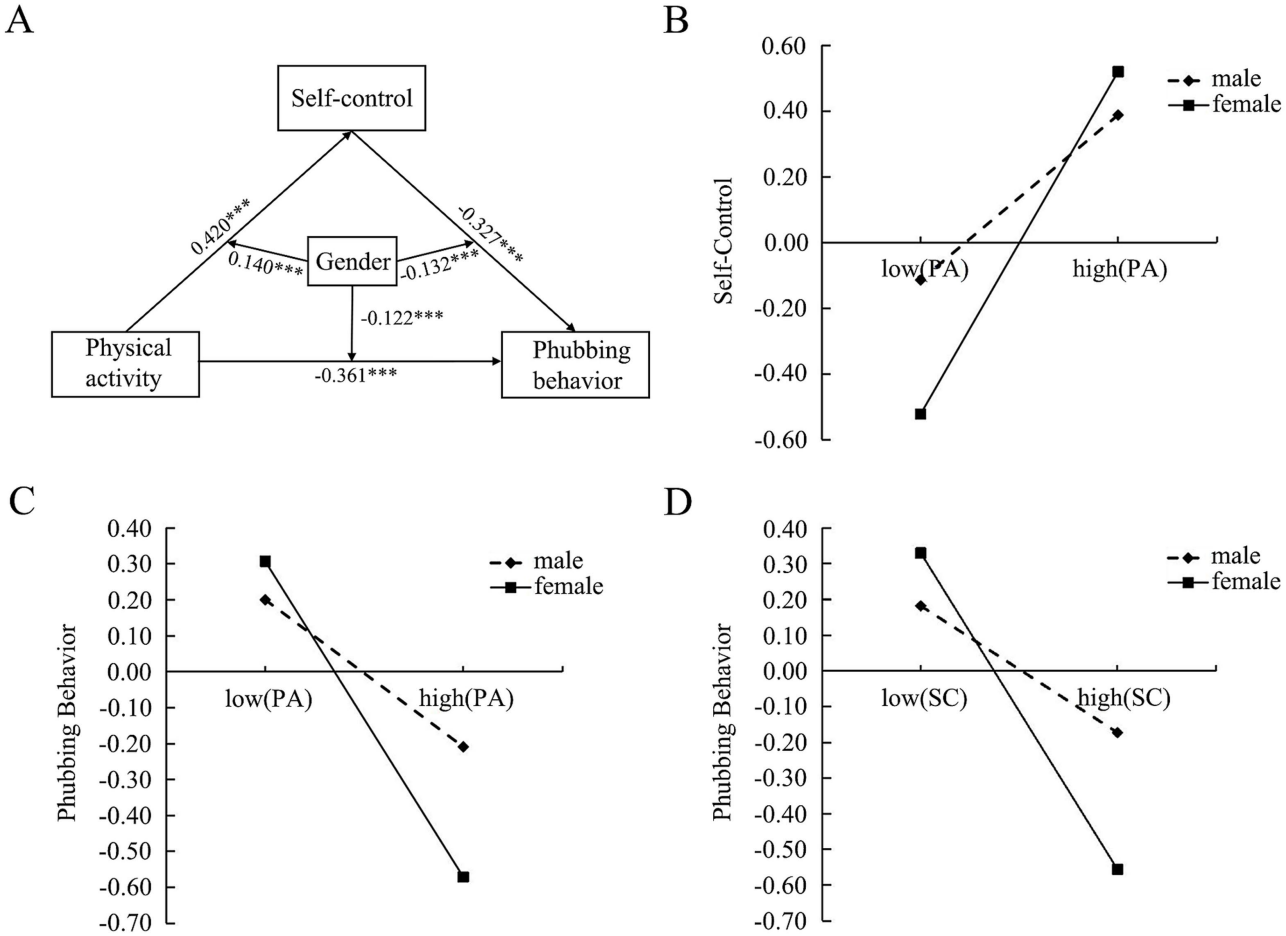
Moderating role of gender. **(A)** Moderated mediation model. ***p < 0.001. **(B)** The moderating effect of gender in the association between physical activity and self-control. **(C)** The moderating effect of gender in the association between physical activity and phubbing behavior. **(D)** The moderating effect of gender in the association between self-control and phubbing behavior.

The analysis of moderating role of gender in model by employing PROCESS (Model 59). As presented in Figure 1A and Table 5. The interaction between physical activity and gender significantly predicted college students’ self-control and phubbing behavior, respectively, (*β* = 0.140, *β* = −0.122, *p* < 0.001), both the interaction between self-control and gender significantly predicted phubbing behavior (*β* = −0.132, *p* < 0.001). Subsequently, to evaluate the statistical significance of conditional indirect effects across different genders by bootstrapping the bias-corrected CI. The analytical outcomes are presented in Table 5 revealed gender-specific variations in mediation patterns. For male participants, the indirect pathway estimate stood at −0.047 [95% CI (−0.073, −0.024)], whereas female counterparts demonstrated a substantially stronger effect magnitude of −0.241 [95% CI (−0.297, −0.190)]. Furthermore, the index was −0.194 [95% CI (−0.256, −0.136)]. These results indicated that gender moderated indirect associations between physical activity and phubbing behavior via self-control, and this mediating role more pronounced among females compared to males. These findings confirmed Hypothesis 3.

**Table 5:** Regression analyses of gender regulation.

Criterion	Predictors	*R*	*R* ^2^	*F*	*β*	SE	*t*
SC		0.407	0.165	52.850***			
	PA				0.420	0.029	14.266***
	If the only-child				0.005	0.026	0.195
	Growth environment				−0.006	0.026	−0.217
	Gender				−0.074	0.027	−2.798**
	Gender × PA				0.140	0.028	5.033***
PB		0.545	0.297	80.601***			
	PA				−0.361	0.029	−11.191***
	SC				−0.327	0.252	−12.992***
	If the only-child				−0.008	0.024	−0.336
	Growth environment				0.010	0.024	0.422
	Gender				−0.058	0.025	−2.370*
	Gender × PA				−0.122	0.028	−4.416***
	Gender × SC				−0.132	0.025	−5.243***

Conditional indirect effect analysis	Effect	BootSE	BootLLCI	BootULCI
Male	−0.047	0.013	−0.073	−0.024
Female	−0.241	0.027	−0.297	−0.191
Index	−0.194	0.030	−0.257	−0.191

PA, Physical Activity; SC, Self-Control; PB, Phubbing Behavior; *p < 0.05; **p < 0.01; ***p < 0.001.

The statistical results of the direct impact of physical activity and the mediation effect of self-control under different genders are presented in Table 6. Subsequently, the study implemented a simple slope analysis explored the interactive effects of physical activity and gender. The analysis indicated that regular physical activity engagement positively correlated with enhanced self-control across both gender groups. Specifically, this predictive relationship maintained statistical significance among female college students (*β* = 0.544, *p* < 0.001) and male college students (*β* = 0.262, *p* < 0.001), and the effect was relatively smaller for male students (Figure 1B). Notably, the analysis indicated that physical activity engagement was a negative predictor of phubbing behavior for female students (*β* = −0.458, *p* < 0.001) and male college students (*β* = −0.213, *p* < 0.001), respectively. However, with elevated physical activity levels, male college students exhibited a less pronounced escalation in phubbing behavior compared to their female college students (Figure 1C). Furthermore, it also demonstrated a significant negative predictive role of self-control on phubbing behavior in female college students (*β* = −0.443, *p* < 0.001) and male college students (*β* = −0.178, *p* < 0.001), however, as self-control increased, male college students’ phubbing behavior reduced more slowly than those of female college students (Figure 1D).

**Table 6:** Effects under different gender conditions.

Total effect	Gender	Effect	BootSE	*t*	*p*-value	BootLLCI	BootULCI
PA–SC	Male	0.262	0.032	8.207	0.000	0.199	0.324
	Female	0.544	0.046	11.830	0.000	0.454	0.634
PA–PB	Male	−0.213	0.031	−6.901	0.000	−0.274	−0.152
	Female	−0.458	0.046	−9.968	0.000	−0.549	−0.368
SC–PB	Male	−0.178	0.038	−4.706	0.000	−0.252	−0.104
	Female	−0.443	0.034	−13.150	0.000	−0.509	−0.377

PA, Physical Activity; SC, Self-Control; PB, Phubbing Behavior.

## 4 Discussion

### 4.1 The influence of physical activity on the phubbing behavior in college students

The current study found that engagement in regular physical activity demonstrated a significant inverse association with phubbing behavior among college students, thereby validating hypothesis 1. The result of present study provided theoretical support for implementing exercise interventions to mitigate phubbing behavior. Phubbing behavior represents a novel social situational behavior that demonstrates significant correlations with individual psychological factors, including loneliness, depression (Bitar et al., 2022; Gao et al., 2024). From the view of basic psychological needs theory, individuals with severe psychological need deficits tend to resort to smartphone usage for need satisfaction, consequently exhibiting phubbing behaviors (Liu et al., 2016). Physical activity not only can effectively reduce loneliness and improve interpersonal relationship quality, on the other hand, it can also reduce the time of smartphone use. Regular physical activity can therefore fulfill college students’ basic psychological needs and reduce the probability of phubbing behavior. Furthermore, to clarify the underlying mechanisms between physical activity and phubbing behavior among college students, the current research specifically investigates the mediating role of self-control within this association.

### 4.2 The mediating role of self-control

The present study positioned self-control as a mediator to examine the underlying mechanism through which physical activity influences college students’ phubbing behavior. Mediation analysis revealed a partial mediation effect, thereby validating hypothesis 2. At same time, some conclusions of this investigation align with established academic research, which confirmed the association between physical activity and self-control capacity as well as verified the significant linkage between self-control and phubbing behavior among college students (Kennett et al., 2009; Chotpitayasunondh and Douglas, 2016). Building upon prior studies, these confirmatory outcomes aligned with existing theoretical frameworks and substantiate the path relationships within our hypothesized model, thereby complementing and expanding the previous research findings. Firstly, the enhancement of self-control capabilities in college students through physical activity can be comprehensively interpreted within the framework of the Self-Control Resource Model (Muraven et al., 2006). This theory posited that self-control relied on finite energy resources that deplete with sustained use. As sustained participation in physical exercise required substantial self-control resources, prolonged engagement leads to significant improvements in individuals’ self-control capacity (Oaten and Cheng, 2011; Zhou et al., 2023). In addition, Evidence from cognitive neuroscience has demonstrated that active participation in physical activities exerts a significant influence on enhancing the functional efficiency of the prefrontal cortex, which is closely associated with executive control processes, thereby creating neurocognitive foundations for improved self-control in technology-mediated social interactions (Berchicci et al., 2013). Those theoretical foundation not only provides a rational explanation for how physical activity enhances college students’ self-control, but also establishes a theoretical foundation for understanding the intermediary mechanism whereby self-control operates between physical activity and the mitigation of phubbing behavior.

Secondly, this study revealed that physical activity exerts an indirect influence on phubbing behavior through the mediating mechanism of self-control in college students, accounting for 30.60% of the total effect. The present study revealed that engagement in physical activity not only directly influenced phubbing behavior, but can also be achieved through the mediating mechanism of self-control. The root cause of maladaptive behaviors lied in deficient self-control capacities, and numerous studies have revealed that enhanced self-control capabilities demonstrate a significant negative correlation with the prevalence of phubbing behavior in interpersonal interactions (Chotpitayasunondh and Douglas, 2016; Jiang et al., 2023). College students’ self-control capacity remain in a developmental phase characterized by instability. When confronted with smartphone temptation, those with greater self-control capacity demonstrate the ability to regulate their behaviors to align with self-expectations and reduce the frequency of smartphone use (Zhao et al., 2024). As a crucial psychological resource, self-control plays a critical role in mitigating phubbing behavior, while active engagement in physical activity serves as a significant pathway for enhancing self-control capacities. Consequently, college students may effectively improve their self-control through regular physical activity, which can contribute to the mitigation of phubbing behavior. The findings of this study indicated that during tertiary education, institutions should not only provide adequate sports facilities and conducive exercise environments, but also actively promote student participation in physical activities. Furthermore, by establishing appropriate fitness goals and fostering self-control development, and can effectively diminish the prevalence and intensity of smartphone-induced phubbing behavior among students.

### 4.3 The moderating role of gender

The present study revealed that moderating influence of gender in the relationship between physical activity, self-control, and phubbing behavior in college students. These findings validated hypothesis 3. Specifically, comparative analysis revealed that female college students’ regular engagement in physical activities exhibited a more pronounced effect on both enhancing self-control and mitigating phubbing behavior when contrasted with their male counterparts.

Gender-specific disparities in physical activity engagement have been extensively examined within academic literature over previous decades. The reasons for this phenomenon may stem from the differences in physiological development and social role expectations between individuals of different genders (Wang Q. et al., 2024; Wang X. et al., 2024). The college stage constitutes a critical developmental juncture between adolescence and emerging adulthood, during which the pressures of employment, academic achievement and interpersonal relationships are particularly prominent. When these pressures cannot be released, students are prone to overuse of smartphone, subsequently leading to phubbing behavior (Samaha and Hawi, 2016). Drawing upon the gender role socialization theory, previous research indicated that female individuals tend to assume greater responsibility in maintaining and nurturing interpersonal relationships. This phenomenon can be attributed to their heightened sensitivity to the emotional needs and social expectations of others, which may consequently lead to demonstrating a higher propensity for phubbing behavior (Forgays et al., 2014; Balta et al., 2018). The current research indicated that female students’ participation in physical activity demonstrates a stronger predictive effect on the enhancement of self-control capabilities. This observed difference could potentially be attributed to variations in engagement in physical activity and phubbing behavior exhibited by the subjects in this study, a conclusion that corroborates findings from prior studies in this domain (Yang et al., 2019; Zhong et al., 2021). While physical activity significantly reduces phubbing behavior among college students, female students demonstrate greater sensitivity to the effects of exercise. This gender-specific difference may be attributed to distinct behavioral response patterns to various exercise modalities, particularly the heightened sensitivity of female students to interpersonal and emotional benefits during physical activity (Coleman et al., 2008). For female college students with low physical activity levels, engagement in physical exercise enhances interpersonal communication, alleviates emotional stress, and improves self-control capabilities. These factors collectively contribute to a more pronounced inhibitory effect on phubbing behavior, resulting in a stronger overall impact of physical exercise on reducing phubbing behavior in female college students compared to male college students. Consequently, these findings suggest that universities should not only provide adequate exercise facilities and encourage active participation in physical activities, but also develop gender-specific exercise strategies to further mitigate the occurrence of phubbing behavior among students.

## 5 Limitations

Although the current findings provide significant insights into the complex interplay among physical activity, self-control, and phubbing behavior through longitudinal investigations, our research is not without its limitations. First, it is important to acknowledge that this investigation primarily employed a cross-sectional design, which inherently limits the capacity to establish causal relationships. Future investigations are recommended to adopt prospective longitudinal study designs, which would enable systematic elucidation of the dynamic causal interplay between physical activity and phubbing behavior through temporal-dynamic observation. Second, although participants were recruited from three universities located in distinct regions of the same province, and implemented rigorous controls for confounding variables (e.g., rural vs. urban locations in China and only-child status) during data processing, the findings’ generalizability to other regions remains constrained. Consequently, it is recommended that future research conduct multi-site replication studies across diverse geographical contexts to validate these results. Finally, the factors influencing phubbing behavior are multifaceted and complex. While self-control was examined in this study, physical activity exerts an indirect influence on phubbing behavior through the mediating mechanism of self-control in college students, accounting for only 30.60% of the total effect, so other psychological indicators (e.g., emotional regulation capacity, anxiety) may also play an important mediating role in this relationship. Therefore, subsequent investigations should prioritize examining these possible variables to enhance the holistic comprehension of the fundamental processes involved.

## 6 Conclusion

The conclusion demonstrates that physical activity constitutes an effective intervention for preventing and reducing phubbing behavior in college students, which can either directly affect college students’ phubbing behavior or indirectly through the mediating variable of self-control. Furthermore, gender influences physical activity, self-control and phubbing behavior in college students, with female students’ physical activity participation exhibiting stronger predictive effects on enhancing self-control and alleviating phubbing behavior compared with male college students.

## Data Availability

The original contributions presented in the study are included in the article/supplementary material, further inquiries can be directed to the corresponding author.
