# Correction: Physical activity and phubbing behavior in Chinese college students: the mediating role of self-control and the moderating role of gender

**DOI:** 10.3389/fpsyg.2025.1665154

**Published:** 2025-09-19

**Authors:** Yue Li, Shanshan Yin, Huijuan Yi, Hao Zhu

**Affiliations:** ^1^Ministry of Public Sports, Taizhou University, Taizhou, China; ^2^Jiangsu Food & Pharmaceutical Science College, Huai'an, China; ^3^School of Physical Education and Humanities, Nanjing Sport Institute, Nanjing, China

**Keywords:** physical activity, phubbing behavior, self-control, college students, moderated mediation

Affiliation School of Physical Education and Humanities, Nanjing Sport Institute was erroneously given as Nanjing Sport Institute, Nanjing University.

There was a mistake in Table 6 as published. The confidence interval data for the PA-PB path in males should be [−0.274, −0.152]. However, in the published Table 6, the negative sign is absent for the value −0.152]. The corrected Table 6 and its caption appear below.

**Table 6:** Effects under different gender conditions.

**Total effect**	**Gender**	**Effect**	**BootSE**	***t***	***p***	**BootLLCI**	**BootULCI**
PA–SC	Male	0.262	0.032	8.207	0.000	0.199	0.324
	Female	0.544	0.046	11.830	0.000	0.454	0.634
PA–PB	Male	−0.213	0.031	−6.901	0.000	−0.274	−0.152
	Female	−0.458	0.046	−9.968	0.000	−0.549	−0.368
SC–PB	Male	−0.178	0.038	−4.706	0.000	−0.252	−0.104
	Female	−0.443	0.034	−13.150	0.000	−0.509	−0.377

PA, physical activity; SC, self-control; PB, phubbing behavior.

In the published article, there was an error in the results description, as the corresponding data for male and female college students were interchanged:

“Specifically, this predictive relationship maintained statistical significance among female college students (β = 0.262, *p* < 0.001) and male college students (β = 0.544, *p* < 0.001).”

A correction has been made to **3 Results**, *3.3.2 Moderating role of gender*, paragraph 2. The corrected sentence appears below:

“Specifically, this predictive relationship maintained statistical significance among female college students (β = 0.544, *p* < 0.001) and male college students (β = 0.262, *p* < 0.001).”

The original version of this article has been updated.

